# Japanese version of the MD Anderson Symptom Inventory for Head and Neck Tumor module: Validation study

**DOI:** 10.1016/j.apjon.2025.100711

**Published:** 2025-05-05

**Authors:** Hiroto Sawaguchi, Masanori Someya, Kensei Nakata, Yu Takada, Keiko Danzuka, Mitsunori Miyashita, Mikiko Kawamura

**Affiliations:** a School of Nursing, Sapporo City University, Sapporo, Japan; b Department of Radiology, Sapporo Medical University School of Medicine, Sapporo, Japan; c Department of Radiology, Sapporo City General Hospital, Sapporo, Japan; d Department of Radiation Oncology, Sapporo Teishinkai Hospital, Sapporo, Japan; e Department of Nursing, Sapporo Medical University Hospital, Sapporo, Japan; f Department of Palliative Nursing, Health Sciences, Tohoku University Graduate School of Medicine, Sendai, Japan

**Keywords:** Head and neck cancer, Patient-reported outcome, Symptom burden, Symptom assessment, Validation, MDASI-HN

## Abstract

**Objective:**

This study aimed to examine the reliability and validity of the Japanese version of the MD Anderson Symptom Inventory Head and Neck Tumor module (MDASI-HN), a patient-reported outcome measure for head and neck cancer.

**Methods:**

The MDASI-HN was translated into Japanese, and cognitive debriefing was conducted. A cross-sectional study was administered to patients with head and neck cancer who were recruited within 5 years of receiving surgery, chemotherapy, or radiotherapy at three cancer treatment centers. The reliability and validity of the Japanese version were confirmed through structural equation modeling, internal consistency, test–retest reliability, convergent validity, known-groups validity.

**Results:**

The Japanese translation of the MDASI-HN was revised with developer feedback. Cognitive debriefing with five patients provided positive feedback regarding the ease of completion and understanding. A cross-sectional sample of 147 patients completed the questionnaire. Structural equation modeling showed a Confirmatory Fit Index of 0.975 and Root Mean Square Error of Approximation of 0.059. The Cronbach's alpha coefficient was 0.88 for head and neck cancer-specific items and 0.96 for all symptom items. The Intraclass Correlation Coefficients (2,1) were 0.72 for HNC-specific items and 0.74 for all items. The convergent validity with the EORTC QLQ-H&N module was *r* ​= ​0.79. The known-groups validity showed small to moderate effect sizes for all subitems, based on the comparison of mean ECOG Performance Status Scale scores between the two groups.

**Conclusions:**

The results showed that the translated MSASI-HN was reliable, valid, and feasible for use in Japanese-speaking patients with head and neck cancer.

## Introduction

In cancer care, careful and ongoing assessment of patient symptoms is essential to the delivery of quality care. Symptom assessment based on patient-reported outcomes (PROs) is critical because it reflects the patient's perspective and helps improve patient care. Recent studies have shown that there are often gaps between patient and clinician assessments, with patient-reported assessments often being more accurate.^1^ Due to the location of the cancer and the characteristics of the treatment, head and neck cancer (HNC) patients experience disease-specific complications and related symptoms.^2^^,^^3^ These factors also affect basic functions such as appearance, speech, and eating, leading to social difficulties.^4^^,^^5^ PROs assess the impact of the disease and treatment from the patient's perspective and offer several advantages for patient care and routine clinical practice.^6^

PROs function as sensitive indicators to evaluate short-term symptoms, accurately reflecting the specific issues experienced by patients and enabling the creation of personalized care tailored to their needs. In addition, PROs play a critical role in patient-centered care. Providing patients with feedback on PRO assessments enhances communication between patients and clinicians, allowing patients to become active participants in their care.^7^^,^^8^ This, in turn, increases patient self-efficacy, improves adherence and health outcomes, and is believed to lead to greater patient satisfaction.^9^ Furthermore, by promoting early intervention, PROs are expected to improve symptom management, increase the likelihood of uninterrupted treatment, and ultimately contribute to better long-term outcomes.^10^

Symptoms in patients with HNC have been assessed using various instruments, including health-related quality of life (QOL) scales such as the European Organization for Research and Treatment of Cancer Quality of Life Questionnaire (EORTC QLQ)-C30 and the EORTC QLQ-Head and Neck Cancer Module (HN35),^11^ as well as symptom assessment tools like the PRO-Common Terminology Criteria for Adverse Events (CTCAE), which can be applied across diseases not limited to the HNC disease-specific symptoms. Implementing a symptom assessment tool, rather than a QOL rating scale, is crucial for improving the accuracy of symptom assessment and promoting effective patient management.^12^ The MD Anderson Symptom Inventory for Head and Neck Tumors module (MDASI-HN) was developed by the MD Anderson Cancer Center (MDACC) and was designed to assess symptoms and their interference with daily activities.^13^ This tool includes 13 core symptom items, six interference items, and nine items specific to patients with HNC. The MDASI-HN enables a direct and accurate assessment of a wide range of unique symptoms experienced by patients with HNC.^3^^,^^12^ However, the MDASI-HN has yet to be validated in Japanese-speaking patients. This study aimed to examine the translation and psychometric validation of the Japanese version of the MDASI-HN in Japanese-speaking patients with HNC. The validation process focused on evaluating the tool's reliability and validity, acceptability to ensure an accurate assessment of symptom burden and interference with daily activities in this population.

## Methods

### Translation and linguistic validation

There is already a Japanese version of the 13 core symptom items and six interference items of the MDASI that is confirmed to be reliable and valid.^14^ After receiving approval from the MDACC Symptom Research Group, a native Japanese speaker, fluent in English, forward translated the original English version of the nine HNC-specific symptom items of the MDASI-HN into Japanese. Two translators who had not seen the original English version then back translated the Japanese into English. The English back translations were reviewed by the MDACC Symptom Research Group and revised several times until the developer found them consistent with the intent and meaning of the original English items.

Five patients who were currently receiving or had recently received treatment participated in the cognitive debriefing assessment of the draft Japanese version of the MDASI-HN. Specifically, each item was evaluated for (1) ease of completion, (2) comprehensibility, (3) acceptability, (4) redundancy, (5) use of scoring system, (6) clarity of items, and (7) confirmation of content domain. Additionally, the time required to complete the questionnaire was recorded. The final version of the MDASI–HN–Japanese was reviewed by the MDACC Symptom Research Group.

### Patient recruitment

The reliability and validity of the Japanese MDASI-HN were confirmed using a cross-sectional sample of patients with HNC. These patients were recruited at three HNC treatment centers: Sapporo Medical University Hospital, Sapporo City General Hospital, and Sapporo Teishinkai Hospital between June 2022 and January 2023. Participants were recruited in outpatient clinics and hospital wards, where they completed the questionnaire on-site and returned it immediately. For the retest, the questionnaire was handed to participants at the time of the initial response and was mailed back by them 3–7 days later. The inclusion criteria were: (1) a diagnosis of head and neck cancer; (2) undergoing or having completed surgery, chemotherapy, or radiation therapy within the past five years; and (3) age 20 years or older; (4) ability to comprehend the questionnaire. The exclusion criteria were: (1) inability to understand Japanese or (2) inability to comprehend the study explanation.

### Instruments

#### Japanese version of MDASI-HN

The MDASI-HN is a 28-item assessment scale that consists of three subscales: 13 core items representing core symptoms common across all cancer types (Core Items), nine HNC-specific items that assess the severity of symptoms related to head and neck cancer (HNC-specific Items), and six items that assess how these symptoms interfere with major activities of the daily life (Interference Items). The MDASI-HN was developed based on a literature review and focus-group input. The Japanese version of the MDASI-HN used the same scoring method as the original English version, where symptom and interference items were rated on scales from 0 (symptom not present or no interference) to 10 (symptom severity as bad as can be imagined or complete interference). Symptom severity and interference at its worst was rated by patients over the past 24 hours.

#### EORTC QLQ-HN35

The EORTC QLQ developed a self-administered questionnaire to assess QOL.^11^ The EORTC QLQ-HN consists of 35 items designed to assess symptoms and difficulties specific to patients with HNC. The reliability and validity of the Japanese version of the EORTC QLQ-HN35 have already been confirmed.^15^ Respondents are asked to rate each item over past week on a 4-point scale from 1 (not at all) to 4 (very much), with higher scores indicating more severe symptoms or greater problems.

#### Other data collection instruments

Participants completed a questionnaire that provided information on gender, age, education level, marital status, cohabitation status, and employment status. In addition, the cancer locations, cancer stage, presence of distant metastases, presence of lymph node metastases, time since last treatment, treatment details, and Eastern Cooperative Oncology Group Performance Status Scale (ECOG PS) were collected from the medical records.

### Statistical analysis

The reliability and validity of the Japanese version of the MDASI-HN were assessed by determining which items should be assessed according to the Consensus-based Standards for the Selection of Health Measurement Instruments (COSMIN)^16^ guidelines and by ensuring that important methodological considerations were adequately addressed for each item.

The sample size was based on previous validation studies. To assess known-groups validity, we conducted a power analysis. Based on an expected effect size of *d* ​= ​0.3, a significance level of *α* ​= ​0.05, and 80% power, the required sample size was 134 participants. We set the target sample size at 150 to account for potential missing data and non-response. This sample size is considered acceptable for structural validity (SEM) because our model has a simple two-factor structure.

Statistical analyses were carried out using SPSS Statistics version 28.0 and IBM SPSS Amos 25. The significance level was set at 0.05, and effect sizes were categorized as small at 0.20, medium at 0.50 and large at 0.80.

The internal structure of the Japanese version of the MDASI-HN was examined through SEM and internal consistency analysis. The SEM compared the factor structure of the Japanese version model with that of the original English version model to evaluate cross-cultural consistency. The SEM not only allows for the evaluation of multiple fit indices but also enables the explicit modeling of interactions between latent variables. Additionally, it facilitates a more detailed examination of the effects of different factor structures on the results while accounting for measurement errors, leading to a more reliable analysis. Considering these advantages, we adopted SEM in this study. Model fit was evaluated using χ^2^, Goodness of Fit Index (GFI), Adjusted Goodness of Fit Index (AGFI), Confirmatory Fit Index (CFI), and Root Mean Square Error of Approximation (RMSEA). Internal consistency was assessed by calculating Cronbach's alpha coefficients for “HNC-Specific Items” and for all items.

The validity of the Japanese version of the MDASI-HN was assessed in terms of known-groups validity and convergent validity. For known-groups validity, it was hypothesized that patients with an ECOG PS of 2–4 would have higher symptom severity scores than those with an ECOG PS of 0–1. Scores were compared using the Mann–Whitney U test, and effect sizes were calculated using Cohen's d. Convergent validity was assessed by calculating Spearman's correlation coefficients between the Japanese version of the MDASI-HN and the EORTC QLQ-HN35-Japanese as an external criterion. A significant positive correlation was expected between the Japanese version of the MDASI-HN and the EORTC QLQ-HN35-Japanese. EORTC QLQ-HN35 is widely used as a measure to assess the QOL in patients with head and neck cancer and was considered an appropriate scale for examining convergent validity.^11^

Test-retest reliability was assessed to evaluate the reliability of the Japanese version of the MDASI. Participants completed the Japanese version of the MDASI-HN again 3–7 days after the baseline assessment. This was to avoid recall bias and to ensure that symptoms had not substantially changed. This interval was determined in consultation with the coauthors to maintain symptom stability. Intraclass correlation coefficients (ICCs) were calculated for the total score and for each item to assess reproducibility. ICCs were computed using the two-way random-effects model ICC (2,1).

## Results

### Translation and linguistic validation

After the original English version of the MDASI-HN was translated into Japanese and then translated back into English version, no notable differences were found compared to the original English version. The review by the MDACC Symptom Research Group included minor comments on three items. Item 14 (Your problem with mucus in your mouth and throat at its WORST?) – The initial back-translation used the phrase “the amount of saliva.” The MDACC group made the point that the question focuses on the severity of problems with mucus, not the amount of saliva or pain. We revised and confirmed the correct translation of “mucus”. Item 16 (Your choking/coughing (food/liquids going down the wrong pipe) at its WORST?) – The back-translation included “trachea”, which is uncommon in English. Since Japanese has a single term for both “trachea” and “pipe”, we modified the translation for consistency. Item 21 (Your mouth/throat sores at their WORST?) – The initial back-translation used “pain” instead of “sore.” Since Japanese does not clearly distinguish between these two terms, we performed another back-translation and confirmed that the Japanese version correctly corresponds to “sore.” The content was then reviewed and retranslated, resulting in final approval by the MDACC Symptom Research Group.

Cognitive debriefing about the Japanese version of the MDASI-HN was conducted with five patients who were either receiving or had received treatment for HNC. The median age of participants was 76 years (range, 59–85 years), and all patients were male. Four participants had an educational level of high school or less. The tumor locations were the larynx (2 patients), nasopharynx (1 patient), oropharynx (1 patient), and parotid gland (1 patient).

All confirmed the ease of completion. All participants found the recall period appropriate. The items were clear and there were no questions that caused discomfort. No redundant items were identified, and four participants reported that no additional items were needed. However, one participant suggested adding an item on “neck tightness due to radiotherapy”. Four of the five participants found the 0–10 scoring system clear. The remaining participant stated that a narrower scoring range would be easier to understand. The median time to complete the questionnaire was 4 minutes (range, 2–7 minutes), with no missing values.

### Collection status and background of the patients

**Fig. 1:**
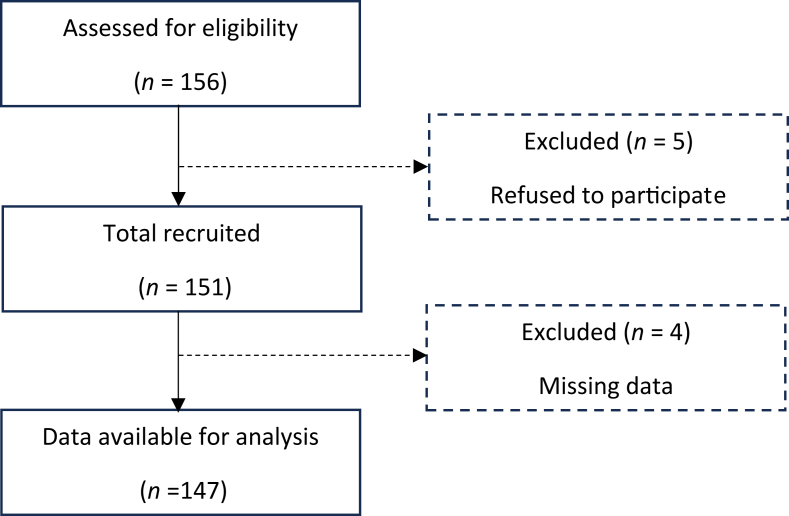
Patient recruitment and analysis flow chart.

The Japanese version of the MDASI-HN was distributed to 156 patients, and responses were received from 151 patients (Fig. 1). Responses with fewer than 14 of the 28 items answered were excluded, resulting in 147 valid questionnaires included in the analysis (valid response rate: 94.2%). The patient characteristics are detailed in Table 1. Among the participants, 78.9% were aged 60 or older, 81.6% were male, and most were post-treatment (87.0%). The disease sites were the larynx (27.9%), oropharynx (21.8%), and hypopharynx (19.7%). For test-retest reliability, the questionnaire was distributed to the initial 147 participants, of whom 127 (86.4%) responded. The score distribution of the Japanese version of the MDASI-HN is summarized by the mean, standard deviation, median, floor and ceiling effects, proportion of scores of 5 or higher, and proportion of scores of 7 or higher (Table 2). Over 80% of the patients reported experiencing at least one symptom. The highest average scores were for “Having a dry mouth”, “Problem with mucus”, “Difficulty swallowing or chewing”, “Problem with tasting food”, “Feeling drowsy” and “fatigue”, in that order. The highest percentage of severe symptom scores indicating a score of 7 or more were “Having a dry mouth” (19.7%), “Problem with mucus” (16.3%) and “Problem with tasting food” (15.0%), “Difficulty swallowing or chewing” (13.6%).

**Table 1:** Characteristics of the participants (*N* ​= ​147).

Characteristics	*n* (%)
**Age (years)**	
< ​60	31 (21.1)
≥ ​60	116 (78.9)
**Sex**	
Female	27 (18.4)
Male	120 (81.6)
**Educational level**	
≤ ​12th grade	94 (63.9)
> ​12th grade	52 (35.4)
Not reported	1 (0.7)
**Marital status**	
Married	125 (85.0)
Single	22 (15.0)
**Living status**	
Living alone	124 (84.4)
Not living alone	23 (15.6)
**Occupational status**	
Employed	54 (36.7)
Unemployed	92 (62.6)
Not reported	1 (0.7)
**ECOG PS**	25 (17.0)
0	93 (63.3)
1	37 (25.2)
2	11 (7.5)
3	6 (4.1)
**Cancer stage**	
I	38 (25.9)
II	30 (20.4)
III	38 (25.9)
IV	39 (26.5)
Unknown	2 (1.4)
**Cancer locations**	
Tongue	11 (7.5)
Oral cavity (excluding the tongue)	10 (6.8)
Nasal cavity	1 (0.7)
Nasopharynx	13 (8.8)
Oropharynx	32 (21.8)
Hypopharynx	29 (19.7)
Larynx	41 (27.9)
Salivary gland	4 (2.7)
Unknown primary site	4 (2.7)
Others	2 (1.4)
**Distant metastasis**	
None	129 (87.8)
Lymph node metastasis	18 (12.2)
**History of anti-cancer treatment**	
Surgery	27 (18.4)
Radiotherapy	58 (39.5)
Chemotherapy and radiotherapy	89 (60.5)
**Time since treatment**	
Ongoing	18 (12.2)
Up to 1 month	20 (13.6)
More than 1 month and up to 1 year	23 (15.6)
More than 1 year and up to 5 years	85 (57.8)
Unknown	1 (0.7)

ECOG PS, Eastern Cooperative Oncology Group Performance Status Scale.

**Table 2:** Descriptive statistics for the Japanese version of the MDASI-HN scores (*N* ​= ​147).

Item	Mean score	SD	Median score	Floor effect (%)	Ceiling effect (%)	% ​≥ ​5	% ​≥ ​7
**Core items**							
Pain	1.3	2.2	0	60.5	0.7	10.9	4.8
Fatigue	1.9	2.3	1	41.5	0.7	16.3	5.4
Nausea	0.8	1.8	0	81.0	0.0	6.8	2.7
Disturbed sleep	1.4	2.2	0	57.8	0.0	11.6	6.1
Distress	1.7	2.3	1	48.3	0.7	15.0	6.1
Shortness of breath	1.4	2.1	0	53.7	0.7	10.2	4.8
Difficulty remembering	1.1	1.8	0	63.9	0.0	7.5	2.0
Lack of appetite	1.6	2.4	0	59.2	0.7	15.0	6.1
Feeling drowsy	2.0	2.3	2	38.8	0.7	15.6	7.5
Having a dry mouth	3.5	2.9	3	22.4	2.7	37.4	19.7
Feeling sad	1.2	2.2	0	64.6	1.4	9.5	4.1
Vomiting	0.8	1.8	0	79.6	0.7	6.8	2.7
Numbness or tingling	1.3	2.2	0	60.5	2.0	11.6	4.1
**HNC-specific items**							
Problem with mucus	3.3	2.7	3	23.1	1.4	36.1	16.3
Difficulty swallowing or chewing	2.7	2.9	2	36.7	2.0	25.9	13.6
Choking or coughing	1.8	2.1	1	42.9	0.7	12.9	3.4
Difficulty with voice or speech	1.9	2.5	1	44.2	1.4	17.7	8.8
Skin pain, burning, or rash	1.2	2.1	0	67.3	0.0	10.2	6.1
Constipation	1.5	2.4	0	56.5	0.0	14.3	8.2
Problem with tasting food	2.4	3.0	1	45.6	3.4	22.4	15.0
Mouth/throat sores	1.7	2.4	0	52.4	0.7	15.6	6.1
Problem with teeth or gums	1.6	2.3	0	54.4	1.4	13.6	6.1
**Interference items**							
General activity	1.5	2.0	0	55.1	0.7	8.2	3.4
Mood	1.5	2.3	0	54.4	1.4	11.6	4.1
Work (including work around the house)	1.5	2.3	0	59.2	1.4	10.9	5.4
Relationships with other people	1.3	2.1	0	61.2	1.4	10.9	3.4
Walking	1.3	2.0	0	59.9	0.7	8.2	3.4
Enjoyment of life	1.7	2.5	0	53.1	1.4	15.6	8.8

MDASI-HN, The MD Anderson Symptom Inventory for Head and Neck Tumors module; SD, Standard deviation; HNC, head and neck cancer. The floor effect: The proportion of responses that selected the minimum value (0 points) among all responses; The ceiling effect: The proportion of responses that selected the maximum value (10 points) among all responses.

### Internal structure evaluation and validity of the Japanese version of the MDASI-HN

**Fig. 2:**
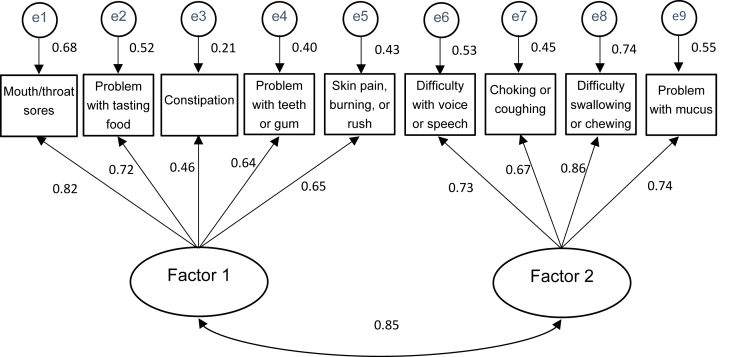
Structural Equation Modeling of nine HNC-specific Items. *n* ​= ​147, χ^2^ ​= ​39.129 (d*f* ​= ​26, *P* ​= ​0.047), Goodness of Fit Index ​= ​0.943, Adjusted Goodness of Fit Index ​= ​0.901, Confirmatory Fit Index, Root Mean Square Error of Approximation, Confirmatory Fit Index of 0.975 and Root Mean Square Error of Approximation of 0.059.

Two factors were identified in the original English version of the nine items of the MDASI-HN ″HNC-Specific Items''.^12^ Similarly, the results of the EFA confirmed the presence of two factors in the HNC-Specific Items of the Japanese version of the MDASI-HN. Subsequently, the SEM was conducted and showed satisfactory model fit indices with a χ^2^ ​= ​39.129 (d*f* ​= ​26, *P* ​= ​0.047), GFI ​= ​0.943, AGFI ​= ​0.901, CFI ​= ​0.975 and RMSEA ​= ​0.059. The path diagram of the covariance structure analysis is shown in Fig. 2.

Internal consistency was assessed and reliability was confirmed with a Cronbach's alpha coefficient value of 0.88 for “HNC-Specific Items” and 0.96 for all items.

Known-group validity was assessed by comparing the mean scores between two groups based on ECOG PS: 0–1 (good) and 2 or greater (poor) (Mann–Whitney *U* test). No statistically significant differences were found in the scores for the “HNC-Specific Items” of the Japanese version of the MDASI-HN for patients with good and poor performance status. Effect sizes calculated with Cohen's d showed small to moderate effects: 0.40 for “Core Items”, 0.21 for “HNC-Specific Items”, and 0.53 for “Interference Items”.

Convergent validity was evaluated by examining the correlation between the Japanese version of the MDASI-HN and the EORTC QLQ-HN35-Japanese. The Spearman correlation coefficient was *r* ​= ​0.79 (*P* ​< ​0.01) for “HNC-Specific Items” and *r* ​= ​0.80 (*P* ​< ​0.01) for “All items”.

### Reliability of the Japanese version of the MDASI-HN

Test-retest reliability was assessed in 127 patients. The ICCs were 0.72 (95% confidence interval 0.62–0.79) for “HNC-Specific Items”, and 0.74 (95% confidence interval 0.65–0.81) for “All items”.

## Discussion

The MDASI-HN was translated from English to Japanese, and its psychometric properties were evaluated. The results confirmed adequate reliability and validity, supporting its applicability for assessing symptom burden in Japanese-speaking patients with HNC.

The results of the cognitive debriefing confirmed that the Japanese version of the MDASI-HN was supported from the patients' perspective. Patients supported aspects such as ease of completion, comprehensibility, acceptability, redundancy, use of the scoring system, and clarity of items. This is a finding that indicates the Japanese version of the MDASI-HN is understandable and useable for patients. On the other hand, for content domain confirmation, the only new item suggested by one participant was “neck tightness due to radiotherapy.” The original development of the MDASI-HN involved thorough validation by experts and patients, with some items removed to focus on the most relevant symptoms applicable across different sites, stages, and treatments.^12^ Although “neck tightness” was removed during the original validation, prior studies have reported a high prevalence of this symptom.^17^ In this study, after sharing information with MDACC Symptom Research Group, the item was not added. The feedback in the present study suggests that this aspect should be considered in future research.

The top-rated symptoms reported in this study were similar to those identified in the original validation of the English version of the MDASI-HN.^13^ However, the mean symptom severity scores were lower than the original validation scores. Most participants were in the post-treatment phase (1–5 years after treatment), when symptoms typically lessen, which may explain the results. Previous studies have reported that symptoms peak at the end of treatment.^3^

Several PROMs are available to assess symptoms and health-related QOL in patients with HNC. A major advantage of using the Japanese version of the MDASI-HN is its ability to comprehensively measure the “symptom burden” specific to HNC. Some PROMs are designed to allow raters to select specific items that they wish to measure. However, symptoms in patients with HNC are not isolated issues but rather clusters of multiple symptoms that affect patients simultaneously.^18^ Thus, the reliable and valid Japanese version of the MDASI-HN provides a significant advantage in assessing overall symptom burden in patients with HNC.

The examination of the internal structure of the Japanese version of the MDASI-HN supported its adequacy. The SEM was conducted and showed satisfactory model fit indices with a CFI of 0.975 and RMSEA of 0.059.^19^ The Cronbach's alpha coefficient and confirmatory factor analysis results indicated that the internal structure was sufficient across languages. Additionally, test-retest reliability demonstrated that it is a reliable scale capable of providing stable measurements. The results showed a reproducibility ICC of 0.72 for “HNC-Specific Items” and 0.74 for “All items”. These values were consistent with those of the original MDASI-HN. In general, a reliability coefficient of 0.70 or higher is considered high, and the values obtained in the present study met this standard.

Two measurement properties, convergent validity and known-groups validity, were evaluated to confirm validity. In terms of convergent validity, a strong correlation of *r* ​= ​0.79 with the EORTC QLQ-HN35 was observed, indicating that the Japanese version of the MDASI-HN shows similar results to the EORTC QLQ-HN35. The results of the known-groups validity showed that, although no statistically significant differences were found, small to moderate effect sizes were observed, suggesting that this does not substantially undermine the validity of the tool. One possible reason for this result is that the number of participants in the ECOG PS 2 or higher group was only 17. This sample size discrepancy may have resulted in finding no statistically significant difference in symptom severity between the two groups. Future research should aim for more balanced sample sizes for reassessment.

### Implications for nursing practice and research

Based on the results of this study, the Japanese version of the MDASI-HN can be considered a useful scale for comprehensively assessing symptom burden in patients with HNC. The MDASI-HN can be considered a useful tool for assessing the symptoms of HNC without burden. This is supported by the high response rates, indicating that patients were able to complete the questionnaire even while experiencing symptoms. The high response rate of 94.2% may suggest that the burden of response was minimal; however, it is also possible that the immediate collection method influenced this result. In addition, the retest response rate was 86.0%, which is relatively high compared with previous studies. This suggests that the burden of response was minimal and that the measure is feasible for use in practice.

Furthermore, more than 80% of participants reported experiencing at least one symptom, with approximately one-third reporting moderate to severe symptoms (scores of 5/10 or higher) for issues such as mucus or dry mouth. Previous studies have similarly identified mucus and dry mouth as common late-onset adverse effects among patients with HNC,^20^ corroborating the present findings. However, given the cross-sectional nature of this study, it may not have fully captured the dynamic aspects of symptom severity over time. Nonetheless, the Japanese version of the MDASI-HN is expected to be a valuable tool for both clinical practice and research. Therefore, to implement the Japanese version of the MDASI-HN in clinical situations, it is necessary to consider how to use the system simply and effectively for both patients and clinicians.^21^

### Limitations

This study has several limitations. First, this study was based on cross-sectional data and did not assess responsiveness to transient symptom changes or changes associated with treatment progression. Future studies should include measurements at appropriate intervals to evaluate responsiveness. Additionally, since understanding the questionnaire was an eligibility criterion, the participants were generally in good condition. There was some potential for participant bias, and further research is needed to evaluate the feasibility of including patients with more severe symptoms. Moreover, there was a lack of data on factors that may influence symptom scores, such as disease stage, treatment modalities, differences in facilities and care, self-care practices, and functional impairments (e.g., presence of laryngectomy or percutaneous endoscopic gastrostomy placement). These factors may affect PROM scores, and future research should address these issues.

## Conclusions

This study assessed the reliability and validity of the translation of the Japanese version of the MDASI-HN, which assesses symptoms in patients with HNC. The results showed that the translation was reliable, valid, and feasible for use in Japanese-speaking patients with HNC.

## Credit authorship contribution statement

**Hiroto Sawaguchi**: Conceptualization, Methodology, Data curation, Formal analysis, Project administration, Writing. **Masanori Someya**: Conceptualization, Supervision, Investigation, Resources. **Kensei Nakata**: Conceptualization, Investigation, Resources, **Yu Takada**: Investigation, Resources. **Keiko Danzuka**: Conceptualization, Investigation, Project administration. **Mitsunori Miyashita**: Methodology, Formal analysis, Writing – Review & Editing, **Mikiko Kawamura:** Supervision, Conceptualization, Methodology, Formal analysis, Writing – Review & Editing. All authors have read and approved the final manuscript.

## Ethics statement

This study was approved by the Research Ethics Committee of the Sapporo City University on 12th January 2022 (Approval No. 10-2) and was conducted in accordance with the 1964 Helsinki Declaration and its later amendments or comparable ethical standards. All participants provided written informed consent.

## Data availability statement

Raw data that support the findings of this study are available from the corresponding author, HS, upon reasonable request.

## Declaration of generative AI and AI-assisted technologies in the writing process

No AI tools/services were used during the preparation of this work, except for basic tools for checking grammar and spelling.

## Funding

This study was supported by the Sapporo City University Graduate Student Research Fund (Hiroto Sawaguchi).

## Declaration of competing interest

The authors declare no conflict of interest.
